# Editorial: Microbial Hydrogen Metabolism

**DOI:** 10.3389/fmicb.2020.00056

**Published:** 2020-01-30

**Authors:** Chris Greening, Eric Boyd

**Affiliations:** ^1^School of Biological Sciences, Monash University, Clayton, VIC, Australia; ^2^Department of Microbiology and Immunology, Montana State University, Bozeman, MT, United States

**Keywords:** hydrogen, hydrogen metabolism, hydrogenase, fermentation, respiration

Among the most ancient and widespread metabolic traits of microbial life is the ability to interconvert molecular hydrogen (H_2_; Lane et al., 2010; Schwartz et al., 2013; Peters et al., 2015). Two classes of metalloenzymes, [FeFe]-hydrogenase and [NiFe]-hydrogenase, catalyze the reversible oxidation of H_2_ to electrons and protons (Volbeda et al., 1995; Peters et al., 1998); a third class of hydrogenase, termed [Fe]-hydrogenase or Hmd, catalyzes the reduction of the substrate methenyltetrahydromethanopterin with H_2_ (Shima et al., 2008). The three classes of enzyme differ structurally and are phylogenetically unrelated. As such, they represent profound examples of convergent evolution (Wu and Mandrand, 1993; Vignais and Billoud, 2007; Greening et al., 2016).

Approximately a third of sequenced microorganisms, spanning at least 70 microbial phyla, encode hydrogenases and are thus predicted to be capable of interconverting H_2_ (Peters et al., 2015; Greening et al., 2016). The earliest evolving hydrogenase enzymes harbor a [NiFe] co-factor and these are thought to have functioned oxidatively (Boyd et al., 2014; Weiss et al., 2016), with [FeFe]-hydrogenases thought to have emerged more recently (Mulder et al., 2010). Both [NiFe] and [FeFe]-hydrogenases have since diversified to function in aerobic and anaerobic, heterotrophic, and autotrophic, and chemotrophic and phototrophic metabolic backgrounds (Kovács et al., 2005; Tamagnini et al., 2007; Thauer et al., 2010; Schwartz et al., 2013; Koch et al., 2014; Schuchmann and Muller, 2014; Pinske and Sawers, 2016). Many bacteria and archaea oxidize H_2_ as a low potential electron donor, an activity typically (albeit not exclusively) attributed to various lineages of [NiFe]-hydrogenase enzymes. Various bacteria, archaea, and microbial eukaryotes also evolve H_2_ as a diffusible end product during fermentative metabolism through the activity of [FeFe]- or [NiFe]-hydrogenases (Horner et al., 2000; Kim and Kim, 2011; Marreiros et al., 2013; Schwartz et al., 2013; Pinske and Sawers, 2016). In many organisms, the ability to metabolize H_2_ is a facultative trait that is regulated through the expression and maturation of hydrogenases (Schwartz et al., 2013; Greening and Cook, 2014). In such taxa, H_2_ represents a substrate that organisms utilize to supplement their energy metabolism, thereby allowing for an expansion of their niche space in ecosystems where other sources of reductant are low or variable in supply (e.g., Amenabar et al., 2018).

The implications of H_2_ in ecosystem level processes is increasingly being realized in both environmental and biomedical settings. A wide range of ecosystems have now been described where H_2_ cycling supports the bulk of primary production and where it forms the basis by which species interact, leading to ecologically structured communities. Much of the research on H_2_ metabolism to date has focused on ecosystems where H_2_ is present at elevated concentrations due to biological activity (e.g., anoxic sediments, gastrointestinal tracts; Sørensen et al., 1981; Wolf et al., 2016; Greening et al., 2019; Kessler et al., 2019) or geological activity (e.g., hydrothermal vents, subsurface systems; Petersen et al., 2011; Brazelton et al., 2012; Telling et al., 2015; Dong et al., 2019; Lindsay et al., 2019). More recently, it has been recognized that atmospheric H_2_ can serve as source of reductant for aerobic soil microorganisms and that this can influence the composition of the atmosphere (Conrad, 1996; Constant et al., 2010; Ji et al., 2017; Cordero et al., 2019). In parallel, medical microbiologists have shown that H_2_ metabolism is critical for the virulence of numerous pathogens, including *Helicobacter*, Clostridia, and Enterobacteriaceae (Kaji et al., 1999; Olson and Maier, 2002; Maier et al., 2004, 2013).

This special issue, featuring 10 articles from 46 different authors, explores microbial H_2_ metabolism from the molecular to the ecosystem scale. In the area of anaerobic metabolism, there are articles exploring the metabolism of H_2_-metabolizing bacteria capable of sulfate reduction, acetogenesis, halorespiration, and fermentation. Two articles investigate H_2_ oxidation in sulfate-reducing bacteria using the model system *Desulfovibrio vulgaris* (Fauque et al., 1988; Caffrey et al., 2007). Smith et al. present a mathematical model of the growth and metabolism of this bacterium, whereas Löffler et al. investigate the kinetic isotope fractionation associated with its H_2_ oxidation activity. A comprehensive review led by Schuchmann et al. covers recent advances in understanding clostridial H_2_ metabolism; it details the discovery and characterization of multimeric electron-bifurcating [FeFe]-hydrogenases, including those associated with formate dehydrogenases (Schut and Adams, 2009; Schuchmann and Müller, 2012, 2013; Buckel and Thauer, 2018). Another article led by Dragomirova et al. focuses on heterologous expression of a [NiFe]-hydrogenase from dehalogenating Chloroflexi (Kublik et al., 2016; Hartwig et al., 2017), reporting another unexpected association with formate dehydrogenase activity. Pinske explores a third type of formate dehydrogenase-linked hydrogenase, namely the classical formate hydrogenlyase complex of Enterobacteriaceae (McDowall et al., 2014), and its association with two novel iron-sulfur proteins.

Several articles also investigate aerobic H_2_ metabolism. Islam et al. report two other novel iron-sulfur proteins in mycobacteria, demonstrating that they are essential for the activity of the two high-affinity hydrogenases described in this lineage (Greening et al., 2014). Carere et al. meanwhile, build on the recent discovery that verrucomicrobial methanotrophs are facultative mixotrophs (Carere et al., 2017; Mohammadi et al., 2017) by showing resource allocation of *Methylacidiphilum* varies depending on H_2_ availability. Three articles also explore H_2_ metabolism at the ecosystem level. Adam and Perner explore the diversity of aerobic and anaerobic H_2_ metabolism in deep-sea hydrothermal vent systems, whereas Meyer-Dombard et al. investigate the influence of H_2_ on biogeochemical cycling in serpentinizing springs in the Philippines. Teng et al. review the previously underexplored area of H_2_ metabolism in bioremediation, including in the reduction of organohalides, nitroaromatic compounds, and heavy metals (Chardin et al., 2003; Hong et al., 2008; Schubert et al., 2018).

In summary, this special Research Topic sheds light on the diverse role of H_2_ in microbial metabolism and uncovers novel enzymes and pathways that mediate this process. This body of work highlights the intricate linkages between H_2_ cycling and the cycling of various other compounds, including methane, formate, carbon dioxide, sulfate, and organohalides, among others. In turn, these findings pave way for future studies on the biochemistry, physiology, ecology, and industrial applications of microbial H_2_ metabolism.

## Author Contributions

CG and EB drafted this editorial together and approve its submission.

### Conflict of Interest

The authors declare that the research was conducted in the absence of any commercial or financial relationships that could be construed as a potential conflict of interest.
